# Supplementary material for: Glycaemic control is associated with SARS-CoV-2 breakthrough infections in vaccinated patients with type 2 diabetes
Source: Nat Commun. 2022 Apr 28;13:2318. doi: 10.1038/s41467-022-30068-2 (PMC9051134; doi:10.1038/s41467-022-30068-2)

Supplementary Information:

Glycaemic control is associated with SARS-CoV-2 breakthrough infections in vaccinated patients with Type 2 diabetes. R. Marfella *et al.*

Supplementary Figure 1. Levels of the one-year mean of HbA1c in subjects with or without infections during the follow-up. The one year mean of HbA1c in patients without infection (n=456) and in patients with infection (n=38). Boxplots show the median, 25th, and 75th percentiles, range, and extreme values. P derives from two-tailed Student's t test.

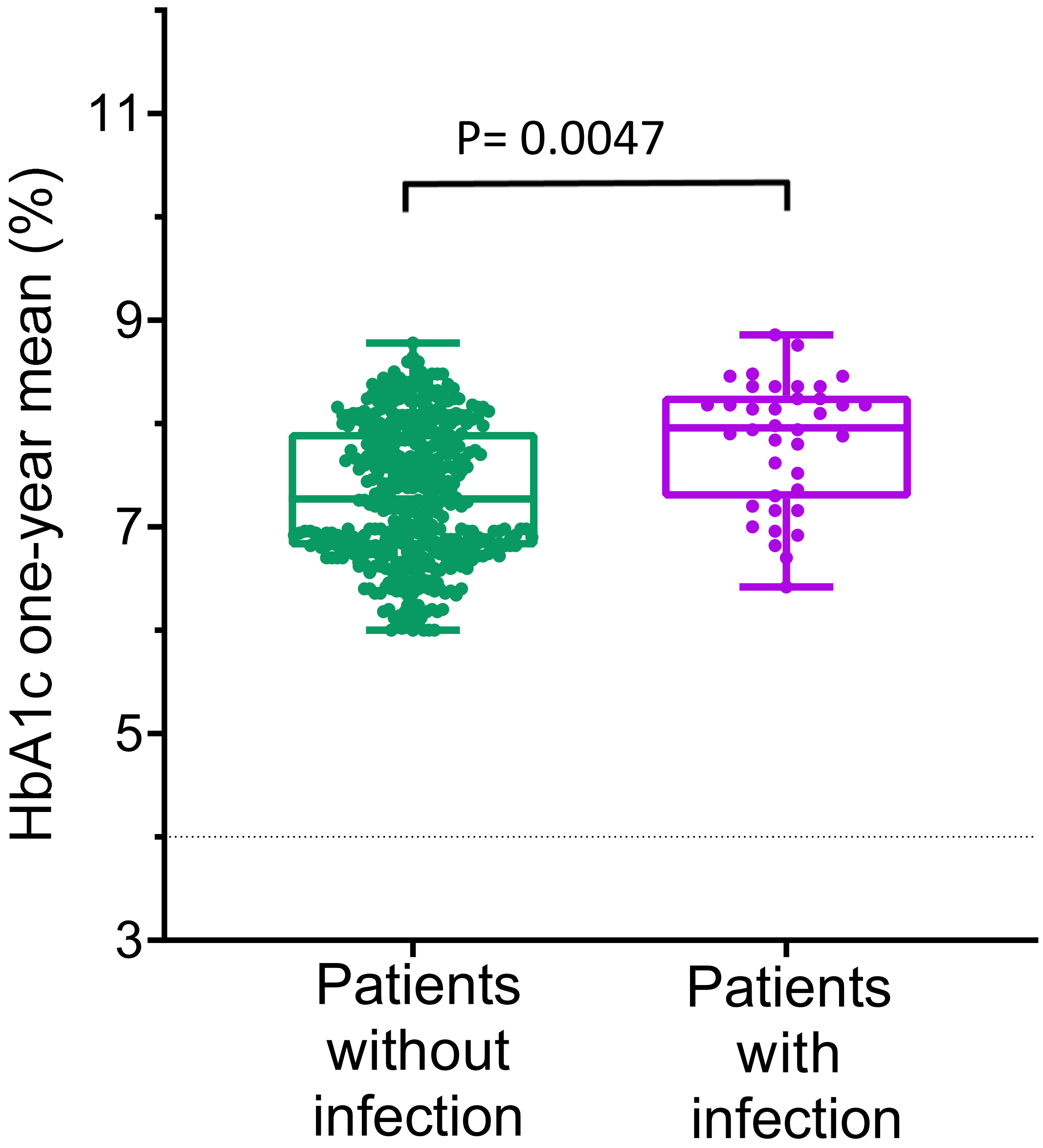

Supplementary Figure 2. Effect of smoking (A) and sex (B) on the development of breakthrough infections. Standardized cumulative incidence of infections comparing smokers vs no-smokers (A) and males vs females (B).

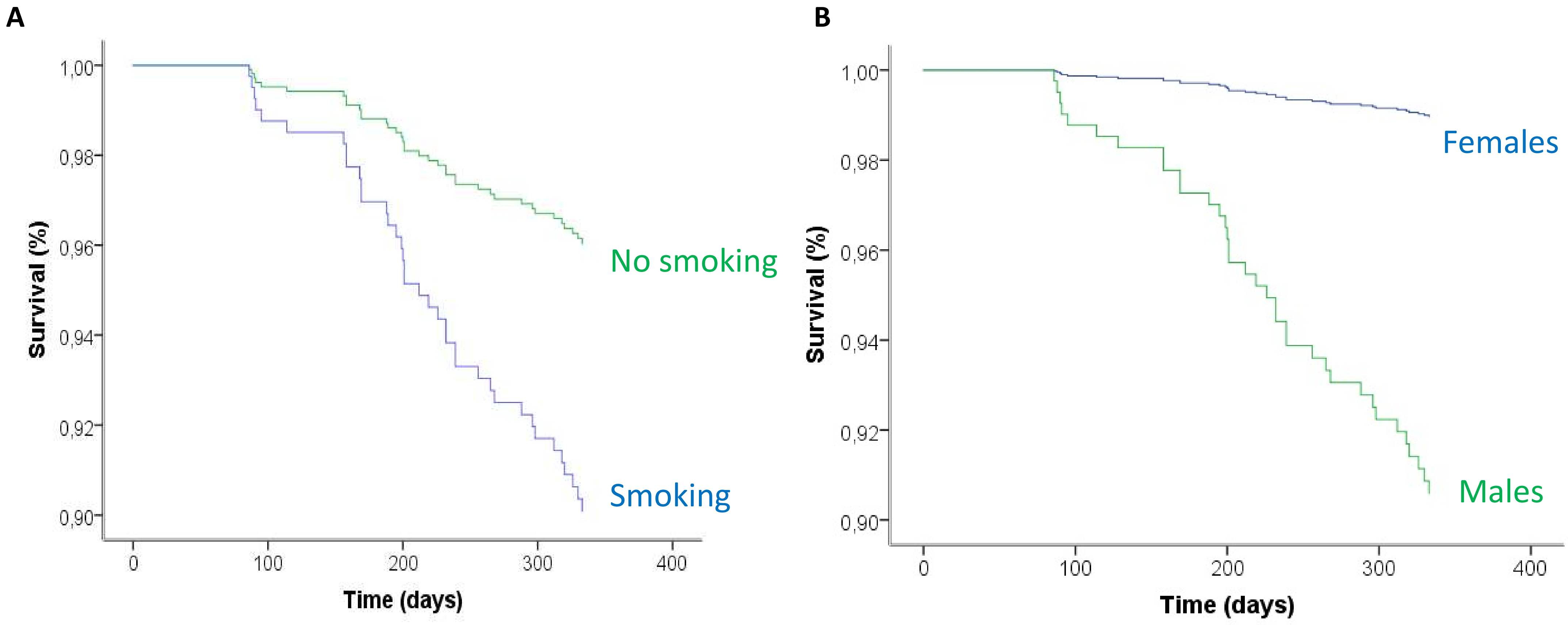

Supplementary Figure 3. Immune-related parameters in subjects with or without infections during the follow-up. The mean of neutralizing antibodies capacity (assessed as percentage of neutralization) (**A**), the proportion of CD4+ T cells producing interferon-γ (IFNγ) (**B**), interleukin-2 (IL-2) (**C**), and tumor necrosis factor- α (TNF-α) (**D**), in subjects without infection (one-year mean, 5 evaluations, n=456), and in patients with infection (mean before infection, 3±2 evaluations, n=38). Boxplots show the median, 25th, and 75th percentiles, range, and extreme values. *P<0.01 Two-tailed Student's t test with Bonferroni correction.

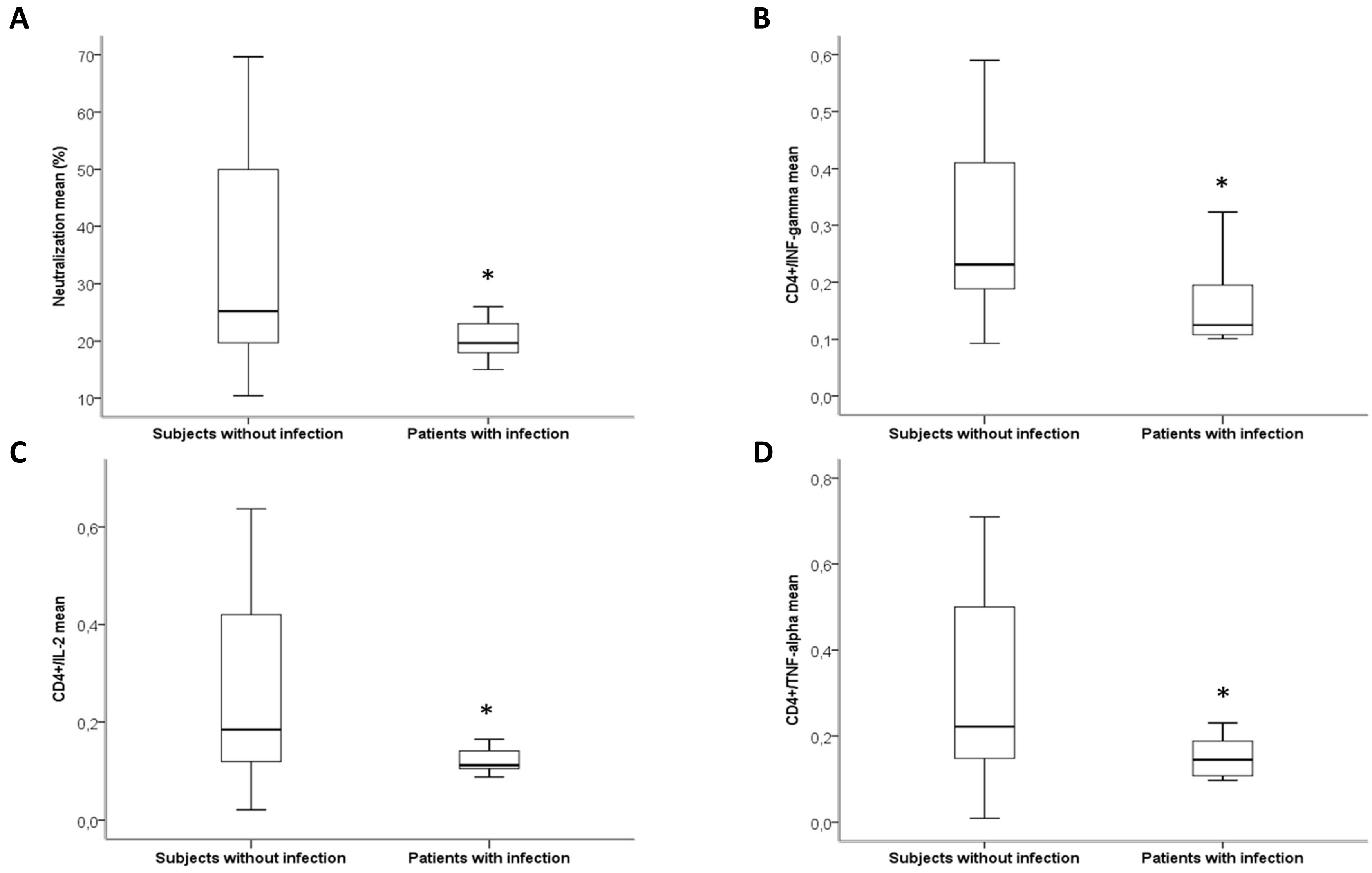

Supplementary Figure 4. Example of the gating strategy used for cytofluorimetric experiments.

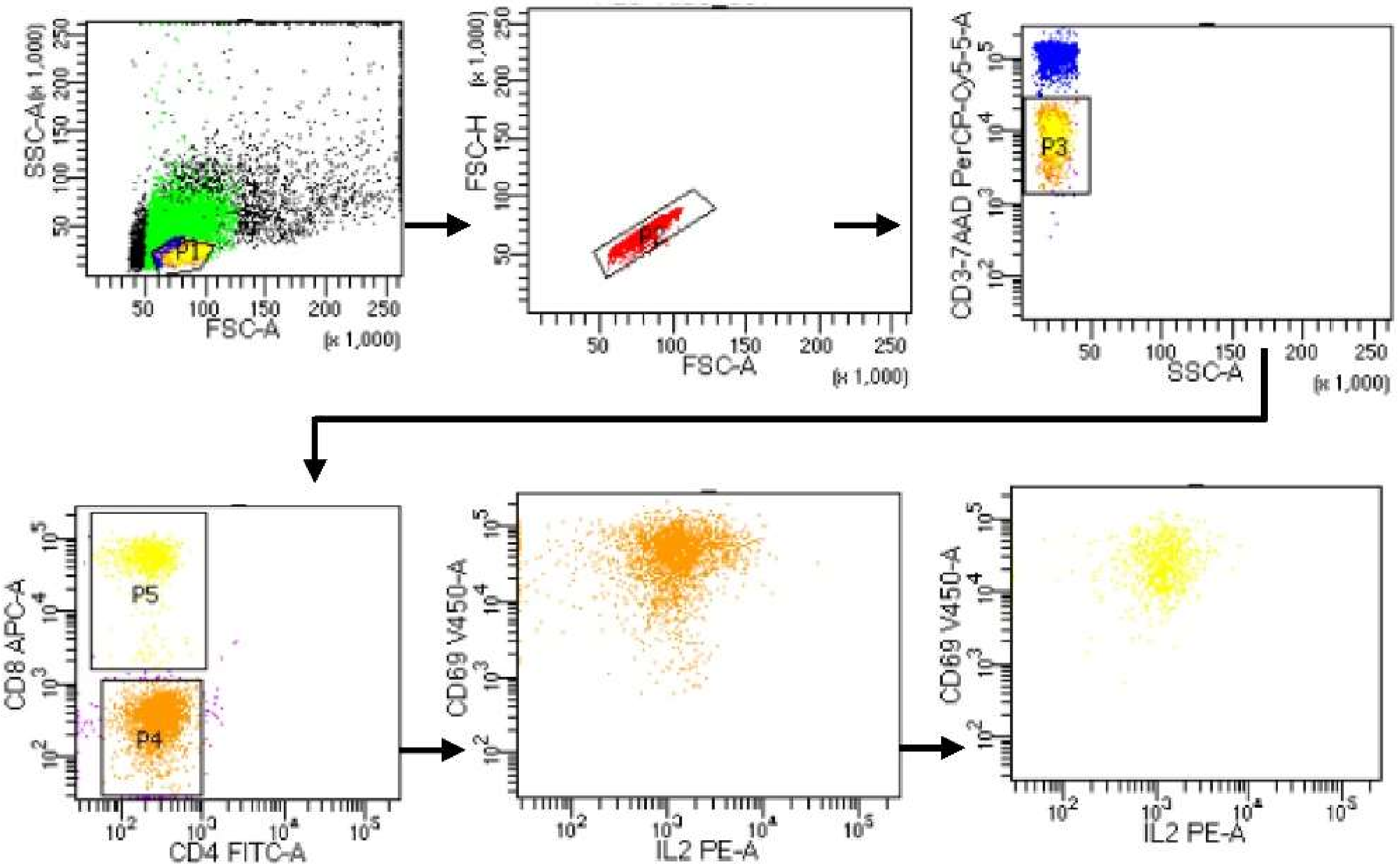

Supplement: Supplementary file 1 — Supplementary Information [file 41467_2022_30068_MOESM1_ESM.pdf]
