# Supplementary material for: Glycaemic control is associated with SARS-CoV-2 breakthrough infections in vaccinated patients with type 2 diabetes
Source: Nat Commun. 2022 Apr 28;13:2318. doi: 10.1038/s41467-022-30068-2 (PMC9051134; doi:10.1038/s41467-022-30068-2)
Supplement: Supplementary file 3 — Reporting Summary [file 41467_2022_30068_MOESM3_ESM.pdf]

nature portfolio

Corresponding author(s): Raffaele Marfella
Antonio Ceriello

Last updated by author(s): Apr 6, 2022

Reporting Summary

Nature Portfolio wishes to improve the reproducibility of the work that we publish. This form provides structure for consistency and transparency in reporting. For further information on Nature Portfolio policies, see our Editorial Policies and the Editorial Policy Checklist.

Statistics

For all statistical analyses, confirm that the following items are present in the figure legend, table legend, main text, or Methods section.

n/a	Confirmed	
☐	☒	The exact sample size (n) for each experimental group/condition, given as a discrete number and unit of measurement
☐	☒	A statement on whether measurements were taken from distinct samples or whether the same sample was measured repeatedly
☐	☒	The statistical test(s) used AND whether they are one- or two-sided<br>*Only common tests should be described solely by name; describe more complex techniques in the Methods section.*
☐	☒	A description of all covariates tested
☐	☒	A description of any assumptions or corrections, such as tests of normality and adjustment for multiple comparisons
☐	☒	A full description of the statistical parameters including central tendency (e.g. means) or other basic estimates (e.g. regression coefficient) AND variation (e.g. standard deviation) or associated estimates of uncertainty (e.g. confidence intervals)
☐	☒	For null hypothesis testing, the test statistic (e.g. F, t, r) with confidence intervals, effect sizes, degrees of freedom and P value noted<br>*Give P values as exact values whenever suitable.*
☐	☒	For Bayesian analysis, information on the choice of priors and Markov chain Monte Carlo settings
☒	☐	For hierarchical and complex designs, identification of the appropriate level for tests and full reporting of outcomes
☒	☐	Estimates of effect sizes (e.g. Cohen's d, Pearson's r), indicating how they were calculated

Our web collection on statistics for biologists contains articles on many of the points above.

Software and code

Policy information about availability of computer code

Data collection	Excel 2019
Data analysis	SPSS 23<br>FlowJo V10

For manuscripts utilizing custom algorithms or software that are central to the research but not yet described in published literature, software must be made available to editors and reviewers. We strongly encourage code deposition in a community repository (e.g. GitHub). See the Nature Portfolio guidelines for submitting code & software for further information.

Data

Policy information about availability of data

All manuscripts must include a data availability statement. This statement should provide the following information, where applicable:

- Accession codes, unique identifiers, or web links for publicly available datasets
- A description of any restrictions on data availability
- For clinical datasets or third party data, please ensure that the statement adheres to our policy

The dataset used for the study has been deposited in Zenodo at 10.5281/zenodo.6412110 and is accessible upon request to raffaele.marfella@unicampania.it.

Field-specific reporting

Please select the one below that is the best fit for your research. If you are not sure, read the appropriate sections before making your selection.

☒ Life sciences ☐ Behavioural & social sciences ☐ Ecological, evolutionary & environmental sciences

For a reference copy of the document with all sections, see nature.com/documents/nr-reporting-summary-flat.pdf

Life sciences study design

All studies must disclose on these points even when the disclosure is negative.

Sample size	Sample size was not calculated before the study. However, our previous study (DOI: 10.1111/dom.14547) showed that even a smaller cohort is sufficient to observe an effect of glycaemic control on immune-mediated parameters. On the other side, studies exploring the determinants of breakthrough infections were generally conducted with larger sample sizes. Thus, the effect of risk factors with small effects might not have been captured here. This aspect has been discussed as a limitation of the study.
Data exclusions	No data excluded
Replication	Two different statistical methods were tested for the main result (Cox and linear regression). Replication was not attempted due to the unique design of the study.
Randomization	No randomization/Observational study
Blinding	PCR testing for SARS-CoV-2 positivity was made by independent laboratories, unaware of the ongoing study. For the other experiments, researchers performing the dosage of immune-mediated parameters were blinded during experiments since the allocation of the groups was post-hoc (glycaemic control was evaluated as the one-year mean of HbA1c at the end of the study).

Reporting for specific materials, systems and methods

We require information from authors about some types of materials, experimental systems and methods used in many studies. Here, indicate whether each material, system or method listed is relevant to your study. If you are not sure if a list item applies to your research, read the appropriate section before selecting a response.

Materials & experimental systems

n/a	Involved in the study	
☐	☒	Antibodies
☒	☐	Eukaryotic cell lines
☒	☐	Palaeontology and archaeology
☒	☐	Animals and other organisms
☐	☒	Human research participants
☒	☐	Clinical data
☒	☐	Dual use research of concern

Methods

n/a	Involved in the study	
☒	☐	ChIP-seq
☐	☒	Flow cytometry
☒	☐	MRI-based neuroimaging

Antibodies

Antibodies used	For flow cytometry analysis, we used the following antibodies: CD4-FITC (#555346, BD biosciences, Clone RPA-T4, 1:100); CD3-PerCP-Cy5-55A (#552851, Clone SP34-2BD biosciences, 1:10); APC-CD8 (# 555369, Clone RPA-T8, BD biosciences, 1:10); V450- CD69 (#560740, Clone FN50, BD biosciences, 1:100); TNF-α-PE (#554513, Clone Mab11, BD biosciences, 1:500); IL-2 –PE (#554566, Clone MQ1-17H12, BD biosciences, 1:500); IFN-γ-PE (#554701, Clone B27, BD biosciences, 1:500).<br>For the assessment of neutralization antibody responses, horseradish peroxidase-conjugated RBD (HRP-RBD, Ref S5-30) was supplied by the kit GenScript SARS-CoV-2 Surrogate Virus Neutralization Test (sVNT) (Cat. No.: L00847-5) and used at the final dilution of 1:50.
Validation	All antibodies used in this study have been validated for human specificity and flow cytometry application, as reported on each specific technical data sheet (FITC Mouse Anti-Human CD4, PerCP Mouse Anti-Human CD3, APC Mouse Anti-Human CD8, V450 Mouse Anti-Human CD69, PE Mouse Anti-Human TNF, PE Rat Anti-Human IL-2, PE Mouse Anti-Human IFN-γ).

Human research participants

Policy information about studies involving human research participants

Population characteristics	Table 1
Recruitment	Participants were recruited among healthcare and educators workers within the Campania vaccination program. Inclusion/ exclusion criteria are described in the methods section. Willingness of patients to participate to the study unlikely introduced a selection bias since the groups of good vs poor glycemic control were composed post-hoc at the end of the study.
Ethics oversight	Ethic committee Università degli Studi della Campania "Luigi Vanvitelli" Protocol n° 0029855

Note that full information on the approval of the study protocol must also be provided in the manuscript.

Flow Cytometry

Plots

Confirm that:

- [x] The axis labels state the marker and fluorochrome used (e.g. CD4-FITC).
- [x] The axis scales are clearly visible. Include numbers along axes only for bottom left plot of group (a 'group' is an analysis of identical markers).
- [x] All plots are contour plots with outliers or pseudocolor plots.
- [x] A numerical value for number of cells or percentage (with statistics) is provided.

Methodology

Sample preparation	Five ml of whole blood was collected in a heparin-coated blood bag and, within an hour of their arrival, centrifuged for 10 min at 1200 rpm to separate the cellular fraction and plasma. Peripheral blood mononuclear cells (PBMC) were isolated using Histopaque-1077 (Sigma Aldrich) by density-gradient sedimentation.<br>Briefly, the blood was layered on cold Histopaque at a 1:1 ratio and subjected to centrifugation at 400×g for 30 min. The white layer containing PBMC was gently aspirated and transferred into sterile centrifuge tubes. After centrifugation, the cell suspension was washed 3 times with PBS and cryopreserved in cell recovery media containing 10% DMSO (GIBCO), supplemented with 10% heat-inactivated foetal bovine serum (FBS, 10270-106, Gibco), and stored in liquid nitrogen until used in the assays. Upon thawing (cell viability > 90%), PBMC were cultured in complete culture medium [(90% RPMI 1640 with 10% Foetal Bovine Serum (FBS) and 1% Penicillin Streptomycin (15140-122, Gibco) and L-Glutamine 25030-024, Gibco)] before intracellular cytokine staining. The mean purity (95-98%) for the PBMC preparation was determined by flow cytometry analysis.
Instrument	FACSAria III (BD Biosciences, San Jose, CA)
Software	FlowJo V10
Cell population abundance	Purity was determined by relevant staining using flow cytometry. At least 10,000 events were originally collected from which positively-gated cells showed 95-98% purity.
Gating strategy	Control stains (unstained and unstimulated samples - single stained cells) were used to set gates. All samples were then FSC-A and SSC-A gated, followed by FSC-A/FSC-H gating to select singlet cells. Subsequent relevant gating was conducted. Specifically, cells were determined by gating on singlets, lymphocytes, CD3+/viability dye followed by CD4+ or CD8+. Each analysed cytokine was plotted vs. CD69, and finally, the double CD69+/cytokine+ events were used as positive responses. Gating for positive cytokines was performed using unstimulated samples. All CD4+ T/CD8+T cells expressing cytokines were reported after background subtraction of identical gates from each individual's negative control stimulation (DMSO).

- [x] Tick this box to confirm that a figure exemplifying the gating strategy is provided in the Supplementary Information.
